# Near-Complete Genome Sequence of a 2019 Novel Coronavirus (SARS-CoV-2) Strain Causing a COVID-19 Case in Peru

**DOI:** 10.1128/MRA.00303-20

**Published:** 2020-05-07

**Authors:** Carlos Padilla-Rojas, Priscila Lope-Pari, Karolyn Vega-Chozo, Johanna Balbuena-Torres, Omar Caceres-Rey, Henri Bailon-Calderon, Maribel Huaringa-Nuñez, Nancy Rojas-Serrano

**Affiliations:** a Laboratorio de Referencia Nacional de Biotecnología y Biología Molecular, Centro Nacional de Salud Pública, Instituto Nacional de Salud, Lima, Peru; b Laboratorio de Referencia Nacional de Virus Respiratorios, Centro Nacional de Salud Pública, Instituto Nacional de Salud, Lima, Peru; DOE Joint Genome Institute

## Abstract

A near-complete genome sequence was obtained for a novel coronavirus (SARS-CoV-2) strain obtained from an oropharyngeal swab from a Peruvian patient with coronavirus syndrome (COVID-19) who had contact with an individual who had returned to Peru from travel to Italy.

## ANNOUNCEMENT

Severe acute respiratory syndrome (SARS) caused by a new coronavirus (CoV), SARS-CoV-2 (genus *Betacoronavirus*, family *Coronaviridae*), initially produced an outbreak of disease in the Wuhan province of China in December 2019; since then, the disease (COVID-19) has spread to different countries on all continents, including South American countries. This situation has led the World Health Organization to declare a global health emergency.

In Peru, more than 28,000 cases have been reported, most of which come from Lima, the capital city of the country (1). To control the disease caused by this new CoV, it is necessary to understand the genetic component of the virus to implement diagnostic methods, new treatments, and vaccines. Here, we report the complete genome sequence of a SARS-CoV-2 strain from a Peruvian patient; the infection was probably acquired from another individual who had travelled to Italy.

For this study, RNA was purified from nasal and pharyngeal swabs from a patient with COVID-19 disease and was amplified using tagged random primers, according to a previously reported protocol (sequence-independent, single-primer amplification [SISPA]) (2). Briefly, first-strand cDNA was synthesized using the K-8N primer and SuperScript III reverse transcriptase (Thermo Fisher Scientific), and then the first-strand cDNA was converted into double-stranded cDNA using Klenow polymerase (Promega). Finally, sequence-independent PCR amplification was conducted using primer K and Platinum *Taq* DNA polymerase, high fidelity (Thermo Fisher Scientific). The DNA obtained was subjected to next-generation sequencing (NGS) using the Nextera XT kit and an Illumina MiSeq sequencer. NGS was performed by the National Reference Laboratory of Biotechnology and Molecular Biology of the Instituto Nacional de Salud, Perú.

The fastq files (2,359,909 reads) were cleaned using Groomer v 1.1.5 and Trimmomatic v 0.38.0 algorithms in the Galaxy platform (3). The reads (2,249,787 paired-end reads) were mapped against the SARS-CoV-2 reference genome (GenBank accession number NC_045512) using the BWA-MEM v 0.7.17.1 algorithm in the Galaxy platform. The reads were assembled using SPAdes v 3.12.0 in the Galaxy platform and were compared to the reference genome using CONTIGuator v 2.7.4 (4). Nucleotide and amino acid variations were detected using the SnpEff v 4.3T program (http://snpeff.sourceforge.net/). Genome sequences reported for SARS-CoV-2 strains belonging to the G, S, or V clade were obtained from the Global Initiative on Sharing All Influenza Data (GISAID) database (https://www.gisaid.org) and aligned using CLUSTAL W v 2.1 (5). Phylogenetic analysis was performed using MEGA X v 10.0.5 (6), using the neighbor-joining algorithm with 1,000 bootstrap replicates. All tools were used with default parameters.

**FIG 1:**
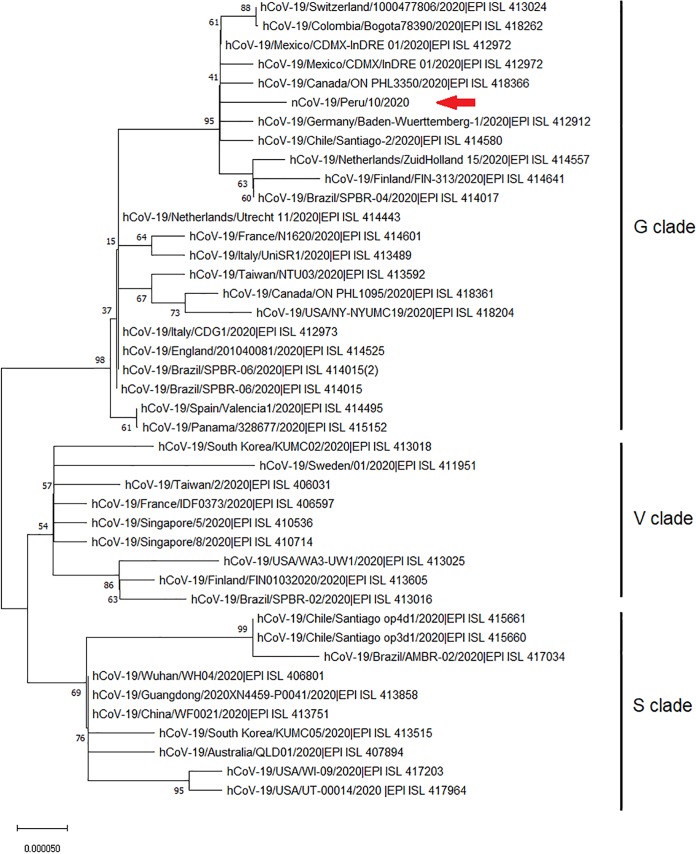
Phylogenetic analysis of genome sequences reported for SARS-CoV-2 strains belonging to the G, S, or V clade, obtained from the GISAID database (https://www.gisaid.org). Phylogeny of the genomes was inferred using the neighbor-joining algorithm with 1,000 bootstrap replicates in MEGA X v 10.0.5. The arrow indicates the location of the genome sequence reported here. The scale at the bottom indicates evolutionary distance, in substitutions per nucleotide.

The near-complete genome of Peruvian SARS-CoV-2 has 29,856 bp, with an average coverage of 84.9×; there were no indels detected. The sequenced genome presents content as follows: 8,915 adenosines (28%), 5,490 cytosines (19%), 5,859 guanines (19%), and 9,592 thymines (34%). Phylogenetic analysis of this virus genome showed that it was grouped in SARS-CoV-2 clade G, which is consistent with that in the other reported cases in South America (Fig. 1).

Analysis of variations indicates few changes in relation to the reference sequence for the Wuhan strain (GenBank accession number NC_045512) from December 2019. We detected a mutation of C to T in noncoding position 25 and other mutations in coding regions that generated amino acid changes such as S1433P, P4720L, and D6909G in the polyprotein encoded by the *orf1ab* gene, D614G in the spike glycoprotein (S), and R203K and G204R in the nucleocapsid protein (N).

We are currently sequencing and analyzing more complete genomes from different regions of Peru to understand the dispersion of the virus and to associate this information with epidemiological data. In this sense, the contribution of SARS-CoV-2 genomes from different countries could facilitate understanding the spread of this virus in South America and worldwide.

### Data availability.

This SARS-CoV-2 genome from Peru was deposited in the international GISAID database (accession number EPI_ISL_415787) and in GenBank (accession number MT263074). The accession numbers for the Illumina MiSeq sequence raw reads in the NCBI Sequence Read Archive (SRA) are PRJNA623683 (BioProject), SRS6448834 (SRA), and SAMN14556477 (BioSample).

## References

[1] Ministry of Health of Peru. Report of cases in Peru. Ministry of Health of Peru, Lima, Peru https://covid19.minsa.gob.pe/sala_situacional.asp.

[2] Chrzastek K, Lee DH, Smith D, Sharma P, Suarez DL, Pantin-Jackwood M, Kapczynski DR 2017 Use of sequence-independent, single-primer-amplification (SISPA) for rapid detection, identification, and characterization of avian RNA viruses. Virology 509:159–166. doi:10.1016/j.virol.2017.06.019. 28646651 PMC7111618

[3] Afgan E, Baker D, Batut B, van den Beek M, Bouvier D, Cech M, Chilton J, Clements D, Coraor N, Grüning BA, Guerler A, Hillman-Jackson J, Hiltemann S, Jalili V, Rasche H, Soranzo N, Goecks J, Taylor J, Nekrutenko A, Blankenberg D 2018 The Galaxy platform for accessible, reproducible and collaborative biomedical analyses: 2018 update. Nucleic Acids Res 46:W537–W544. doi:10.1093/nar/gky379. 29790989 PMC6030816

[4] Galardini M, Biondi EG, Bazzicalupo M, Mengoni A 2011 CONTIGuator: a bacterial genomes finishing tool for structural insights on draft genomes. Source Code Biol Med 6:11. doi:10.1186/1751-0473-6-11. 21693004 PMC3133546

[5] Thompson JD, Higgins DG, Gibson TJ 1994 CLUSTAL W: improving the sensitivity of progressive multiple sequence alignment through sequence weighting, position-specific gap penalties and weight matrix choice. Nucleic Acids Res 22:4673–4680. doi:10.1093/nar/22.22.4673. 7984417 PMC308517

[6] Wisecaver JH, Hackett JD 2014 The impact of automated filtering of BLAST-determined homologs in the phylogenetic detection of horizontal gene transfer from a transcriptome assembly. Mol Phylogenet Evol 71:184–192. doi:10.1016/j.ympev.2013.11.016. 24321593

